# Kidney injury molecule-1 and microalbuminuria levels in Zambian population: biomarkers of kidney injury

**DOI:** 10.11604/pamj.2016.24.54.8759

**Published:** 2016-05-13

**Authors:** Mildred Zulu, Trevor Kaile, Timothy Kantenga, Chisanga Chileshe, Panji Nkhoma, Musalula Sinkala

**Affiliations:** 1 University of Zambia, School of Medicine, Department of Pathology and Microbiology, Kalundu, Lusaka, Zambia; 2 University Teaching Hospital, Department of Pathology and Microbiology, Kalundu, Lusaka, Zambia; 3 University of Zambia, School of Medicine, KS-HHV8 Research and Diagnostic Laboratory, Kalundu, Lusaka, Zambia; 4 University of Zambia, School of Medicine, Department of Biomedical Sciences, Kalundu, Lusaka, Zambia

**Keywords:** Kidney injury molecule-1, microalbumin, creatinine, urea, kidney disease

## Abstract

**Introduction:**

Kidney injury affects renal excretion of plasma analytes and metabolic waste products with grave pathologic consequences. Early detection, thus of kidney injury is essential for injury specific intervention that may avert permanent renal damage and delay progression of kidney injury. We aimed to evaluate Kidney Injury Molecule-1 (KIM-1) and Microalbuminuria (MAU), as biomarkers of kidney injury, in comparison with creatinine.

**Methods:**

We compared the levels of urine MAU, urine KIM-1 and other plasma biochemical tests in specimens from 80 individuals with and without kidney disease.

**Results:**

We found no difference in KIM-1 levels between the kidney disease group (2.82± 1.36ng/mL) and controls (3.29 ± 1.14ng/mL), *p* = 0.122. MAU was higher in participants with kidney disease (130.809± 84.744 µg/mL) than the controls (15.983± 20.442µg/mL), *p* ?0.001. KIM-1 showed a weak negative correlation with creatinine (r = -0.279, p = 0.09), whereas MAU was positively correlated with creatinine in participants with kidney disease with statistical significance (r = 0.556, *p* = 0.001).

**Conclusion:**

The study demonstrated that in Zambian setting MAU and creatinine are sensitive biomarkers in the diagnosis of kidney damage. We moreover propose further evaluation of KIM-1 as a biomarker of kidney injury.

## Introduction

Kidney injury is a condition in which the kidneys fail to function adequately. It is described as a decrease in the glomerular filtration rate [1]. Kidney malfunction frequently culminates to abnormal fluid levels in the body, deranged acid levels, abnormal levels of electrolytes, haematuria and anaemia [1, 2]. There are two forms of renal injury; acute kidney injury (AKI) and chronic kidney disease (CKD), which are indicative of the rate at which damage occurs, rather than the mechanisms by which it occurs [3]. It has been postulated that introduction of therapy early in the disease process would reduce the mortality rate associated with AKI and also delay the progression of CKD to end stage kidney disease (ESKD) [4]. The identification of reliable biomarkers for kidney injury would be useful to facilitate early intervention, evaluate the effectiveness of therapeutic interventions, and guide pharmaceutical development by providing an indicator of nephrotoxicity [4] The standard methods of assessing renal function have kept the measurement of serum blood urea nitrogen and creatinine, biomarkers that are insensitive and nonspecific, especially in the setting of AKI [5, 6]. Serum Creatinine levels are affected by non-renal factors such as age, gender, race, muscle mass, protein intake and liver function [7, 8], whereas blood urea nitrogen is affected by protein intake, dehydration, liver function, gastrointestinal bleeding and steroid use [9]. Increase in creatinine levels is seen when 60-75% of nephrons are non-functional while increase in urea is seen when approximately 75% of the nephrons become malfunctional, and therefore, these markers are not reliable for the diagnosis of kidney damage [10].

It is also important to recognize that changes in serum creatinine and blood urea nitrogen concentrations primarily reflect functional changes in filtration capacity and are not true ‘injury markers’ [11]. There is a crucial need for better biomarkers of AKI for its timely diagnosis, for the prediction of severity and outcome and for the monitoring of proximal tubule injury in AKI but also for progression in chronic kidney disease (CKD) [12].

Kidney injury molecule-1 (KIM-1) is a Type I transmembrane structural glycoprotein with a cleavable ectodomain (90 kDa) which is located in the apical membrane of dilated tubules [13, 14]. It is minimally expressed in healthy kidneys, but is rapidly up-regulated and expressed on the apical surface of renal epithelial cells of the proximal tubule in response to different forms of renal injury [13, 15]. It plays a role in limiting the autoimmune response to injury by phagocytosis of apoptotic bodies and other debris from the tubular lumen [16, 17]. Presence of KIM-1 in urine has been found to be highly specific for kidney injury because no other organ has been shown to express KIM-1 to a degree that would influence kidney excretion [9]. Tissue KIM-1 expression and urinary KIM-1 levels have been reported to be increased in renal damage models of experimental animal studies and in several human studies [8, 18]. However there is paucity of data on the levels of KIM-1 in Zambian patients with kidney disease

Microalbuminuria (MAU) is defined as the subclinical elevation of urinary albumin, predominantly due to abnormal urine excretion of albumin between 30 and 300µg/ml [19]. It has been described as a biomarker of kidney damage, end stage renal disease (ESRD) and a risk of developing cardiovascular disease. It has also emerged as a strong candidate in the prediction of renal risk in hypertensive and diabetic patients, and may signal the presence of functional and / or structural renal abnormalities that precedes and envisage the onset of GFR deterioration [19, 20]. The pathophysiological mechanisms underlying the presence of MAU are still not clear, though intra renal hemodynamic changes that are brought about by increased systemic blood pressure, or capillary leakiness at the glomerular level have been implicated with the latter reflecting a more generalized atherosclerotic vascular damage [20, 13].

Currently, kidney injury is typically diagnosed by measuring serum creatinine and blood urea nitrogen [5, 6]. However, these biomarkers are insensitive for the diagnosis of early kidney damage [10]. Due to the diagnostic shortfalls of current routine biomarkers for evaluation of early kidney damage, various novel biomarkers have been suggested for assessment of kidney injury, with KIM-1 being among the front-runners based on the prospects it has as a biomarker as shown in various studies. Here, we evaluated KIM-1 and MAU as biomarkers of kidney damage in patients with kidney disease in the Zambian population. Ethical Approval Consent & Permissions: The University of Zambia Biomedical Research Ethics Committee (UNZABREC) IRB00001131 of IORG0000774, approved the study protocol; approval reference No. 003-08-14. Written Informed consent was obtained from all study participants. Permission to conduct the study was obtained from the Directorate of Research and Graduate Studies (DRGS) of the University of Zambia and from the Medical Supretendant of the University Teaching Hospital of Zambia (UTH).

## Methods

**Study design, site and sampling methods:** An analytical cross-sectional study was conducted at the UTH in Lusaka, Zambia, involving 40 individuals with confirmed kidney disease (cases) and 40 individuals without any past or present history of kidney disease (controls), from whom consecutive blood and urine specimens were collected. The two groups were matched for broad age categories and sex.

**KIM-1 Estimation:** KIM-1 concentration was determined using the NeoBioLab^®^Human HK0032 (United States) ELISA Kit; a quantitative competitive immunoassay for measurement of Human KIM-1 in urine according to the manufacturer's protocol. **MAU Estimation**- MAU concentration was determined using the Fitzgerald^®^ Industries International (United States) ELISA Kit, a quantitative competitive immunoassay for measurement of Human albumin in urine. **Renal functional test estimation (creatinine, urea and electrolytes (Sodium, Potassium and Chloride)** - were measured using the Beckman Coulter Olympus AU480 automated chemistry analyser.

**Data analysis:** The independent student's t-test was used to compare mean values of urinary KIM-1, urinary MAU, creatinine, urea and electrolytes and Pearson's correlation was used to assess for correlation. All statistical tests were performed at 5% significance level and differences were considered significant if 2-tailed p < 0.05.

## Results

**Figure 1:**
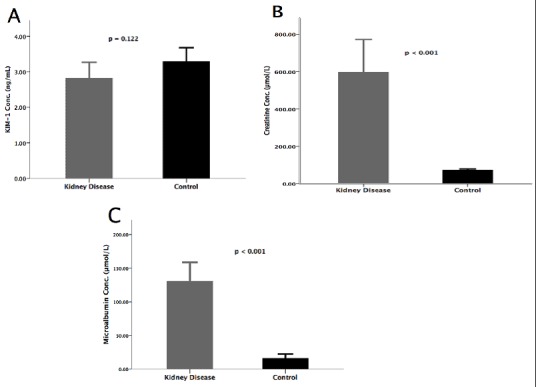
A) Mean difference of KIM-1 Levels: KIM-1 levels by ELISA in 40 participants with kidney disease and 40 participants without kidney disease; B) mean difference of microalbumin levels: microalbumin levels by ELISA in 40 participants with kidney disease and 40 participants without kidney disease; C) mean difference of creatinine levels: serum creatinine levels by ELISA in 40 participants with kidney disease and 40 participants without kidney disease

There was no difference in KIM-1 concentration between the cases (participants with kidney disease) (2.82 ± 1.36ng/mL) and controls (participants without kidney disease) (3.29 ± 1.14ng/mL), *t*(69) = -1.565, p <0.122 (Figure 1 A). MAU concentration and creatinine concentration were significantly higher in the cases 130.8 ± 84.7µg/mL and 597.9 ± 553.3µmol/L respectively, than in controls 16.0 ± 20.4µg/mL and 72.4 ± 17.7µmol/L respectively, with statistical significance, t(75) = 8.316, p ?0.001 and t(74) = 5.930, p?0.001 respectively (Figure 1 B and Figure 1 C, respectively).

**Figure 2:**
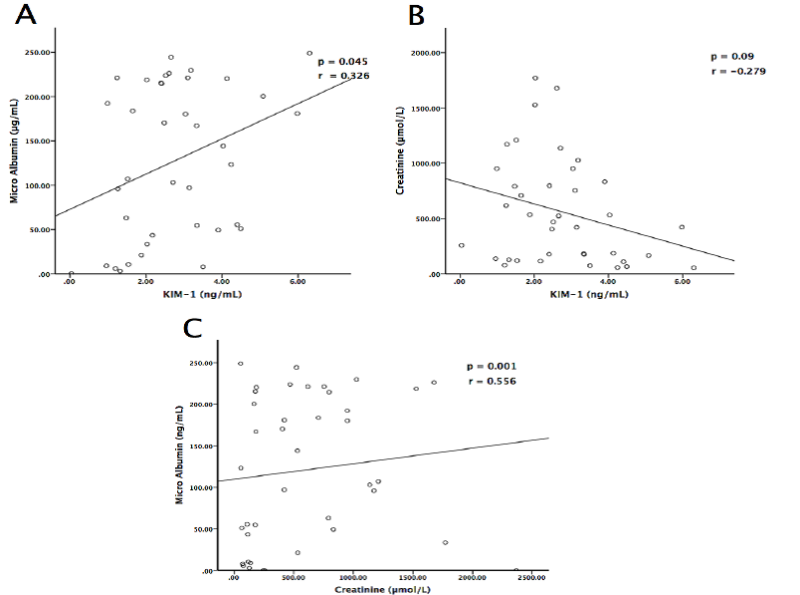
Correlations of microalbumin, creatinine and KIM-1 in participants with kidney disease: A) microalbumin showed a weak positive correlation to KIM-1 with statistical significance; B) serum creatinine showed a non-significant weak positive correlation to KIM-1; C) microalbumin showed a moderate positive correlation to creatinine with statistical significance

KIM-1 showed a weak positive correlation to MAU in participants with kidney disease with statistical significant r = 0.326, p = 0.045 (Figure 2A), however KIM-1 showed a weak negative correlation to creatinine in participants with kidney disease without statistical significance r = -0.279, p = 0.090 (Figure 2 B). MAU showed a medium positive correlation to creatinine in participants with kidney disease with statistical significance, r = 0.556, p = 0.001 (Figure 2 C).

## Discussion

Our results showed that there was no difference in KIM-1 concentration between participants with kidney disease and those without (Figure 1 A). These results were inconsistent with other studies that found elevated KIM-1 levels in participants with renal disease [4, 10, 14, 15, 18]. The disparity in these findings could be attributed to pathophysiological changes of the kidney due to the drug treatment effect [15]. According to a study done by Seo et al (2013) [16], they found lowered KIM-1 levels after renal drug treatment [16]. They accordingly proposed that urinary KIM-1 may be a useful biomarker for evaluating and predicting tubulointerstitial repair; hence an indicator of response to treatment [12, 15]. These findings could be interpreted in line with our results based on the similarity that our study participants were also on renal drug treatment.

The probable role of KIM-1 as a biomarker of kidney injury might be explained in part by a weak positive correlation between urinary KIM-1 and MAU with statistical significance (Figure 2 A) These results were similar to other studies which showed a positive correlation between KIM-1 with MAU, serum creatinine and blood urea nitrogen (BUN) [7, 17]. Therefore, an increase in KIM-1 level could be associated with a rapid decline in renal function [17]. However, this positive correlation did not hold between KIM-1 and creatinine (Figure 2 B), demonstrating that unlike KIM-1 levels, which may decrease due to treatment effect, creatinine levels may continue to increase as renal function deteriorates [15]. Participants with kidney damage, nevertheless, had significantly higher levels of creatinine (Figure 1 B), urea, potassium and chloride, and significantly lower sodium levels (Table 1). These results were characteristic of kidney injury with a decline of renal function in the group with kidney disease [17, 21]. Specifically, failure of the kidneys to excrete urea and creatinine, and increased loss of potassium with impaired sodium reabsorption in kidney disease [22]. Furthermore, the group with kidney disease showed significantly higher MAU concentration than the control group (Figure 1 C). Increased MAU/ albuminuria signal an increased risk of death, cardiovascular disease and kidney disease progression [23, 24]. MAU has emerged as a strong candidate in the prediction of renal risk in hypertensive as well as diabetic patients, it has been proposed that this condition may signal the presence of functional and / or structural renal abnormalities that precede and predict the onset of GFR deterioration [25]. Our results demonstrate the importance of MAU as a biomarker of kidney injury in participants with kidney disease, which is further justified by the moderate positive correlation between MAU and creatinine with statistical significance (Figure 2 C). These results are similar to other studies, which reported a positive correlation between MAU and creatinine in impaired kidney function [26].

**Table 1:** Clinical and biochemical mean difference between participants with kidney disease and controls without kidney disease

Variable	Kidney disease	No kidney disease	*P*
Urea (mmol/L)	18.8 ± 14.71	4.0 ± 1.72	< 0.001
Sodium (mmol/L)	135.9 ± 4.78	138.7 ± 2.16	< 0.001
Potassium (mmol/L)	4.54 ± 0.76	4.16 ± 0.58	0.016
Chloride (mmol/L)	106.0 ± 5.12	102.4 ± 2.79	< 0.001

Urea, Potassium and Chloride were higher in participants with kidney disease than in controls without kidney disease. Sodium levels were lower in kidney disease participants than in controls. P values calculated using independent sample t test.

## Conclusion

Our study showed that there was no difference in KIM-1 levels between participants with kidney disease and those without kidney disease. MAU levels and serum creatinine levels were consistently higher and positively correlated in participants with kidney disease. Thus, the finding shows that MAU and serum creatinine should be the biomarkers of choice for the diagnosis and monitoring of renal damage. However, prospective studies are needed to investigate the role of KIM-1 in predicting kidney injury or its reversibility following treatment.

### What is known about this topic

- Kidney injury molecule-1 (KIM-1) expression can persist until the damaged cells have completely recovered;
- Though insensitive to early kidney damage, urea and creatinine are currently the biomarker of choice in the diagnosis of kidney injury;
- Following renal injury, there are increased levels of intact albumin in the urine.

### What this study adds

- Unlike other studies which have indicated that KIM-1 is not present in normal kidneys, this study showed that it is actually present;
- The study also showed that currently creatinine and urea are still better biomarkers in the diagnosis of kidney damage compared to KIM-1 and that microalbuminuria is a better biomarker of kidney damage than KIM-1;
- The study also adds to the Zambian pool of scientific knowledge in that no other studies have determined KIM-1 levels in the Zambian population.
